# Habitat‐related seed germination traits in alpine habitats

**DOI:** 10.1002/ece3.3539

**Published:** 2017-11-24

**Authors:** Maria Tudela‐Isanta, Eduardo Fernández‐Pascual, Malaka Wijayasinghe, Simone Orsenigo, Graziano Rossi, Hugh W. Pritchard, Andrea Mondoni

**Affiliations:** ^1^ Department of Earth and Environmental Sciences University of Pavia Pavia Italy; ^2^ Department of Biological Sciences George Washington University Washington DC USA; ^3^ Comparative Plant and Fungal Biology Royal Botanic Gardens Ardingly UK; ^4^ Department of Agricultural and Environmental Sciences University of Milan Milano Italy

**Keywords:** alpine zone, dormancy, functional traits, germinability, germination strategies, microhabitats, phylogenetic least squared regression

## Abstract

Understanding the key aspects of plant regeneration from seeds is crucial in assessing species assembly to their habitats. However, the regenerative traits of seed dormancy and germination are underrepresented in this context. In the alpine zone, the large species and microhabitat diversity provide an ideal context to assess habitat‐related regenerative strategies. To this end, seeds of 53 species growing in alpine siliceous and calcareous habitats (6230 and 6170 of EU Directive 92/43, respectively) were exposed to different temperature treatments under controlled laboratory conditions. Germination strategies in each habitat were identified by clustering with k‐means. Then, phylogenetic least squares correlations (PGLS) were fitted to assess germination and dormancy differences between species’ main habitat (calcareous and siliceous), microhabitat (grasslands, heaths, rocky, and species with no specific microhabitats), and chorology (arctic–alpine and continental). Calcareous and siliceous grasslands significantly differ in their germination behaviour with a slow, mostly overwinter germination and high germination under all conditions, respectively. Species with high overwinter germination occurs mostly in heaths and have an arctic–alpine distribution. Meanwhile, species with low or high germinability in general inhabit in grasslands or have no specific microhabitat (they belong to generalist), respectively. Alpine species use different germination strategies depending on habitat provenance, species’ main microhabitat, and chorotype. Such differences may reflect adaptations to local environmental conditions and highlight the functional role of germination and dormancy in community ecology.

## 1 INTRODUCTION

Alpine ecosystems harbor plant communities that grow above the natural tree‐line and are the only bioclimatic zone found in all latitudes of Earth (Nagy & Grabherr, 2009). These high elevation environments present challenges for plant life, including exposure to strong winds, large temperature fluctuations (from freezing to extreme heat events), short growing seasons, and usually nutrient‐poor soils. Despite being subjected to extreme conditions, alpine zones contain 4% of higher vascular plant species with a high level of endemism (Körner, 2003).

In the European highest massifs, such as the Alps, snow‐protected grasslands dominated by sedge heath and dwarf shrubs are typical (Ozenda & Borel, 2003), although there is a clear landscape contrast between calcareous and siliceous bedrocks. In each bedrock, different environmental filters, for instance the soil physical and chemical parameters (i.e., Ca^2+^ or Al), have strong effects on species composition. Within each bedrock type, there is a mosaic of microhabitats occupied by different plant communities, from shelter scan vegetation to tussock grasses (Körner, 2003). In this context, understanding the key aspects of plant regeneration from seed is crucial to assess plant mechanisms to the alpine habitats (Fernández‐Pascual, Jiménez‐Alfaro, & Díaz, 2013).

Germination is an irreversible process and must be timed to occur when the environment is favorable for subsequent seedling establishment (Poschlod et al., 2013; Thompson & Fenner, 2005). Germination timing is controlled by environmental cues (Lambers, Stuart Chapin, & Pons, 2008), chiefly an obligate requirement for soil moisture and variable temperature inputs (Probert, 2000). Dormancy, in addition to germination cuing, will determine germination timing, therefore, the likelihood of seedling survival. Indeed, some species are inhibited to germinate until they have received the appropriate cue such as light or fire (Baskin & Baskin, 2014). Responses to these cues have evolved into specific germination strategies and dormancy states that reflect species adaptations to different habitats (Willis et al., 2014) and trigger germination at favorable sites and times for seedling establishment. Even, the conditions experienced by the mother plants, during flowering and dispersal time, will in turn influence the germination timing of the following generation (Burghardt, Metcalf, Wilczek, Schmitt, & Donohue, 2015). For instance, maternal soil nutrient levels are known to affect the offspring phenotypic expression (Wulff, Causin, Benitez, & Bacalini, 1999), such that, when mother plants were grown under high nitrogen concentrations weaker dormancy status of the offspring were identified.

Examples of direct environmental features driving variation in germination strategies include habitat disturbance (Angevine & Chabot, 1979), altitude (Fernández‐Pascual, Jiménez‐Alfaro, & Bueno, 2017), soil nutrients (Hilhorst & Karssen, 2000), density of plant cover (Jankowska‐Blaszczuk & Daws, 2007), and chorology (Orsenigo et al., 2015). Despite the strong environmental control on plant regenerative traits (e.g., dispersal vectors, seed longevity, germination), they still remain underrepresented when studying the drivers of vegetation patterns in local and global scales (Kleyer et al., 2008). However, seed traits (Pierce, Bottinelli, Bassani, Ceriani, & Cerabolini, 2014) and particularly germination traits (i.e., timing and degree of germination) may play an important role in promoting species coexistence within communities (Jiménez‐Alfaro, Silveira, Fidelis, Poschlod, & Commander, 2016; Kos & Poschlod, 2008). For example, seed weigh had been found to be related to seed longevity in the soil seed bank (Cornelissen et al., 2003) and with the species’ competitive ability (Tilman, 1994).

In the alpine environment, the large species and microhabitats diversity have resulted in a variety of germination responses and dormancy types, which makes it difficult to define a common “alpine” germination strategy (Hoyle et al., 2015; Körner, 2003; Schwienbacher, Navarro‐Cano, Neuner, & Erschbamer, 2011). For example, although many alpine plants have deep physiological dormancy (Baskin & Baskin, 2014; Schwienbacher et al., 2011; Sommerville, Martyn, & Offord, 2013) and require light (Jaganathan, Dalrymple, & Liu, 2015) and high temperatures for germination (Jumpponen, Vare, Mattson, Ohtonen, & Trappe, 1999), nondormant seeds (Sommerville et al., 2013), very low temperature requirements and dark conditions (Schwienbacher et al., 2011) for germination have also been observed.

The steep environmental gradients (e.g., temperature and water) found within a few meters in the alpine habitat (Graham et al., 2012) provide an ideal context to assess changes in germination strategies related to the local environment. Indeed, differences in germination traits have been attributed to slope orientation (Xu, Li, Zhang, Liu, & Du, 2017), biogeographical provenance (Giménez‐Benavides, Escudero, & Pérez‐García, 2005), and species’ successional niche (Schwienbacher, Navarro‐Cano, Neuner, & Erschbamer, 2012). The high variability germination responses identified in alpine plants have been ascribed as a survival strategy to face unpredictable environmental conditions (Kigel, 1995). Therefore, an investigation at habitat level may help clarifying germination patterns in alpine species and add further insights into their functional significance in community ecology. To this end, here we conducted germination experiments with 53 species inhabiting in the two most representative alpine habitats in Europe, namely those on siliceous (26 species) and calcareous (27 species) bedrocks (European Commission, 2007), using a combination of different pre‐treatments (i.e., cold stratification and GA_3_) and incubation temperatures (i.e., 25/15°C and 15/5°C). We hypothesized that each habitat would be dominated by different germination behaviors related to local environmental variables; for instance, species’ inhabiting in calcareous habitats will show lower germinability when dispersal than those from siliceous habitat because risk of drought is high in the former (Gigon, 1942). To test this, we assessed whether (1) in siliceous and calcareous habitats, species show common germination strategies and (2) germination traits can be influenced by the species’ main microhabitat (grasslands, rocky, heaths, and species occurring in more than one microhabitats) and by their chorology (arctic–alpine and continental) (sensu Passalacqua, 2015).

## 2 MATERIALS AND METHODS

### 2.1 Study system

The studied species belong to the Natura 2000 habitat types “6230—*Nardus*‐rich species grasslands”; and “6170—Alpine and subalpine calcareous grasslands” (92/43/CEE “Habitat” Directive classification) (European Commission, 2007). Habitat #6230 occurs in nutrient‐poor soils with acidic pH, which increase the availability of ions like A^l+^ and H^+^. This habitat is formed on various types of siliceous rocks (mainly crystalline slides and granite, and volcanic rock). Its frequent plant species include *Nardus stricta*,* Carex curvula*, and *Arnica montana* (Gennai, Foggi, Viciani, Carbognani, & Tomaselli, 2014). Meanwhile, habitat #6170 is characterized by nutrient‐rich soils and alkaline pH, which increase the availability of ions like Ca^2+^. It is formed on calcareous bedrocks and its typical plant species include *Sesleria caerulea*,* Dryas octopetala*, and *Phyteuma orbiculare* (European Commission, 2007). Calcareous habitats are drier than siliceous because of their lower water holding capacity (Körner, 2003). Moreover, calcareous soils hold a higher biodiversity than siliceous in which many endemisms, rarities, and species with high biogeographical value occur (Pawlowsky, 1970). For simplicity, each habitat is referred to hereafter by its soil type (siliceous and calcareous) or code (#6230; #6170).

### 2.2 Germination experiments

Fifty‐three species, representing 19 plant families, were chosen based on their occurrence and abundance in these two habitats. From the 53 species included in this research, 26 species were collected in the siliceous habitats and 27 were from the calcareous bedrocks. Inside each habitat, different microhabitats such as rocky, grasslands, or heaths places were identified; for this reason, species were collected also having in account their main microhabitat. Species’ chorology and microhabitat were derived from Aeschimann, Lauber, Moser, and Theurillat (2004) (Table S1). Chorology (sensu Passalacqua, 2015) was considered as the area of distribution of the species, while microhabitat was defined as the most common place for species occurrence: grasslands, rocky, heaths, and generalist (i.e., species occurring in more than three microhabitats).

Freshly harvested matured seeds were collected from about 50 to 100 plants of each of the 53 species at time of natural dispersal (Hay & Smith, 2003) in 2015 (i.e., August to October) in the following Sites of Community Interest (SCI): Val Viola Dosde (46°24′N, 10°12′E) and Passo dello Stelvio (46°32′N, 10°25′E), both located in the Alps of Lombardy (Sondrio, northern Italy). From each, about 20–100 seeds were collected depending on the species. After collection, seeds were cleaned, pooled, and stored at room temperature until the beginning of the experiments, which occurred within 2 weeks after the collection. This methodology was chosen because we were interested in defining seeds’ primary dormancy and to avoid any possible change in the germination and/or dormancy response induced by holding seeds in the laboratory (Baskin & Baskin, 2014). Indeed, after ripening had different effect on species’ germination increasing the final germination percentage for *Avena fatua* (Johnson & Dyer, 2000) or decreasing it for *Eucalyptus pauciflora* (Beardsell & Mullett, 1984).

Laboratory experiments involved subjecting sown seeds to three cold stratification periods of 0, 3, and 5 months (hereafter referred to as 0, 3, and 5 CS) at 0°C in complete darkness. 0°C was chosen as cold stratification temperature because it represents mean temperatures registered at the study area (Mondoni, Rossi, Orsenigo, & Probert, 2012); snow cover buffers soil temperatures in alpine habitats avoiding temperatures to reach extremely freezing values. All germination tests started the same day. After each interval, seeds were incubated for germination at two alternating temperatures to simulate summer (25/15°C) and autumn/spring (15/5°C) daily field conditions, reflecting conditions during the most suitable period for seedling emergence at the species growing sites (Mondoni et al., 2012). For each species and treatment, three samples of 20 seeds each were sown on 1 % distilled water‐agar in 50‐mm‐diameter Petri dishes. Following the Baskin and Baskin seed dormancy classification (2014), one of the important distinguishing features of the degree of physiological dormancy is whether seeds respond to gibberellins (GA_3_). Consequently, seeds were also incubated at 25/15°C with 250 mg/L of GA_3_ incorporated into 1% agar. Plates were checked for germination monthly during the cold stratification and weekly (for 5 weeks) during the germination at 25/15 and 15/5°C. Seeds were scored as germinated when the radicle protruded >2 mm. At the end of the experiments, non‐germinated seeds were cut‐tested to confirm their viability. Empty seeds or fungus infected with fleshy or dark embryo were considered non‐viable. Then, the final germination percentage (FGP) and the time to 50% of germination (*T*_50_) were calculated excluding non‐viable seeds. All germination tests were carried out in temperature and light‐controlled incubators (LMS 250A; LMS Ltd, Sevenoaks, UK) using a 12‐h daily photoperiod (photosynthetically active radiation 40–50 μmol m^−2^ s^−1^). The results of the experiments were used to create a germination matrix in which each species was assigned the 13 or 15 germination outputs (see Table S2), representing all the species’ mean (or GA_3_ aside) FGP and *T*_50_ of each treatment. *T*_50_ of each treatment was calculated using R version 3.3.2. The log‐logistic, a dose‐response model, is fitted to the cumulative germination data to calculate the time needed to reach 50% of germination from the total viable seeds.

### 2.3 Habitat‐related seed germination traits

The treatments (i.e., including 0 CS, 3 CS, 5 CS, and GA_3_ at 25/15°C and 15/5°C) were used to assess differences in the FGP between habitats (i.e., siliceous and calcareous). In these models, mean FGP of each species and treatment with a logit transformation were fitted with different phylogenetic least squared regression (PGLS) (Grafen, 1989) implemented in the package “nlme” (Pinheiro, Bates, DebRoy, & Sarkar, 2015) against habitat. PGLS was used because it incorporates an expected model of evolution and phylogeny into the variance‐covariance matrix (Kraft et al., 2015). This accounts for the non‐independence among observations (i.e., species) due to closely related species having similar traits values (Harvey & Pagel, 1991). The phylogenetic signal (λ) and the regression parameters were calculated simultaneously by a maximum‐likelihood (ML) estimation (Revell, 2010). The value of λ ranges from 1 to 0. High values of λ indicate phylogenetic dependence among observations as predicted by a Brownian evolution model, whereas values close to 0 indicate phylogenetic independence among observations.

Moreover, germination strategies were assessed within habitats using cluster analysis computed with the Euclidean distance matrix using the 15 germination outputs (i.e., *T*_50_ and FGP) and k‐means algorithm from the Factoextra package in R (Kassambara, 2015). The appropriate number of cluster was chosen considering the results of different combinations of number of clusters, distances measure, and clustering methods in both habitats with the package NbClust (Charrad, Ghazzali, Boiteau, & Niknafs, 2015). Finally, four germination strategies within each habitat were compared and plotted using two principal component analysis (PCA) with the “FactoMineR” package (Le, Josse, & Husson, 2008).

### 2.4 Seed dormancy classes

To assign each species under a seed dormancy class (sensu Baskin & Baskin, 2014), information related to seed coat permeability and embryo type was obtained from the literature (Baskin & Baskin, 2007; Martin, 1946). To determine physiological and morphophysiological dormancy level, generalized lineal mixed models (GLMM) with binomial error structure and logit link function were built for each species. In these models, seed germination proportion (i.e., number of germinated seeds out of number of viable seeds of each species) was the response variable, whereas dormancy‐breaking treatment [four‐level categorical variable including 0 CS, 3 CS, 5 CS, and GA_3_], temperature (two‐level categorical variable, including 15/5, 25/15°C), and their interaction were the explanatory variables. Finally, replicates were treated as a covariable. According to the data collected from the literature and the germination response observed here, species were assigned a type and class of dormancy following the Baskin and Baskin classification (2014) and Silveira's diagram Silveira (2013). For example, when the FGP of seeds incubated at 3 or 5 CS were significantly higher than FGP at 0 CS, the embryo was fully developed at dispersal and the seed coat was permeable, seeds were considered PD (for further information about the classification criteria used see Table S3a,b).

### 2.5 Phylogenetic comparative analysis

We conducted a comparative phylogenetic analysis to evaluate the influence of microhabitat and chorology on species germination behavior. First, the germination matrix, in which 13 germination outputs were included (i.e., FGP and *T*_50_ for all treatments with GA_3_ aside), was reduced using a new principal component analysis (PCA). Then, species scores in the PCA Axis I and Axis II were regressed against the species chorology and microhabitat using PGLS. Finally, the best model to explain each axis was selected using Akaike information criterion (AIC) (Burnham, Anderson, & Huyvaert, 2011).

### 2.6 Seed weight

The weight of 50 seeds (g) collected during the growing season 2016 was measured. Mean seed weight for each species was obtained from five replicate weights. Weight was log‐transformed to proceed with statistical analysis. Firstly, correlations between seed weight and Axis I, Axis II, FGP 0M 15/5°C, and FGP 0M 25/15°C were assessed fitting lineal models. Secondly, differences on species’ weight between habitats and microhabitats were compared using PGLS.

## 3 RESULTS

### 3.1 Habitat‐related seed germination traits

Seed germination varied across habitats for some treatments (Table 1). The PGLS revealed that species from the siliceous habitat had a significantly higher FGP than those from calcareous habitat in the 0 CS and 15/5°C treatment, although no differences in FGP were found between habitats in the other treatments (i.e., 0 CS and 25/15°C, 3 CS and 5 CS and 15/5°C, 3 CS and 5 CS 25/15°C, GA_3_). Further analysis showed that FGP during cold stratification was higher in species from the calcareous habitat (i.e., 12%) compared to those from the siliceous (i.e., 6%), although in each habitat, 14 and 12 species (i.e., calcareous and siliceous habitat, respectively) germinated during the cold stratification (i.e., 0°C and darkness) (see Fig. S1).

**Table 1:** Results from the phylogenetic least squares relation between habitat and final germination percentage

Response variable	λ	FGP in calcareous grassland (%)	FGP in siliceous grassland (%)	*p* Value
FGP during cold stratification	**1.00**	**12**	**6**	**<.001**
FGP‐0 CS 25/15	0.67	43	46	.67
FGP‐0 CS 15/5	**1.02**	**14**	**23**	**<.001**
FGP‐3 CS 25/15	0.56	51	60	.65
FGP‐3 CS 15/5	0.43	57	57	.95
FGP‐5 CS 25/15	0.70	87	88	.14
FGP‐5 CS 15/5	0.37	58	59	.63
FGP‐GA3	0.28	57	93	.53

Significant (*p* < .05) values in bold character.

The habitat–PCA results were similar. The Axis I was explaining 54.7% and 51.3% of the total variance for calcareous and siliceous habitat, respectively. Axis I was correlated with all treatments, positively with the FGP and negatively with *T*_50_ summarizing capacity of germination in both habitats. Axis II explained 14.2% and 17.2% of the variance for calcareous and siliceous habitats, respectively. It was mainly positive correlated with FGP 0 CS in the calcareous habitat and with FGP 0 CS and FGP during cold stratification in the siliceous. Axis II was explaining the capacity of the species to germinate right after dispersal.

**Figure 1:**
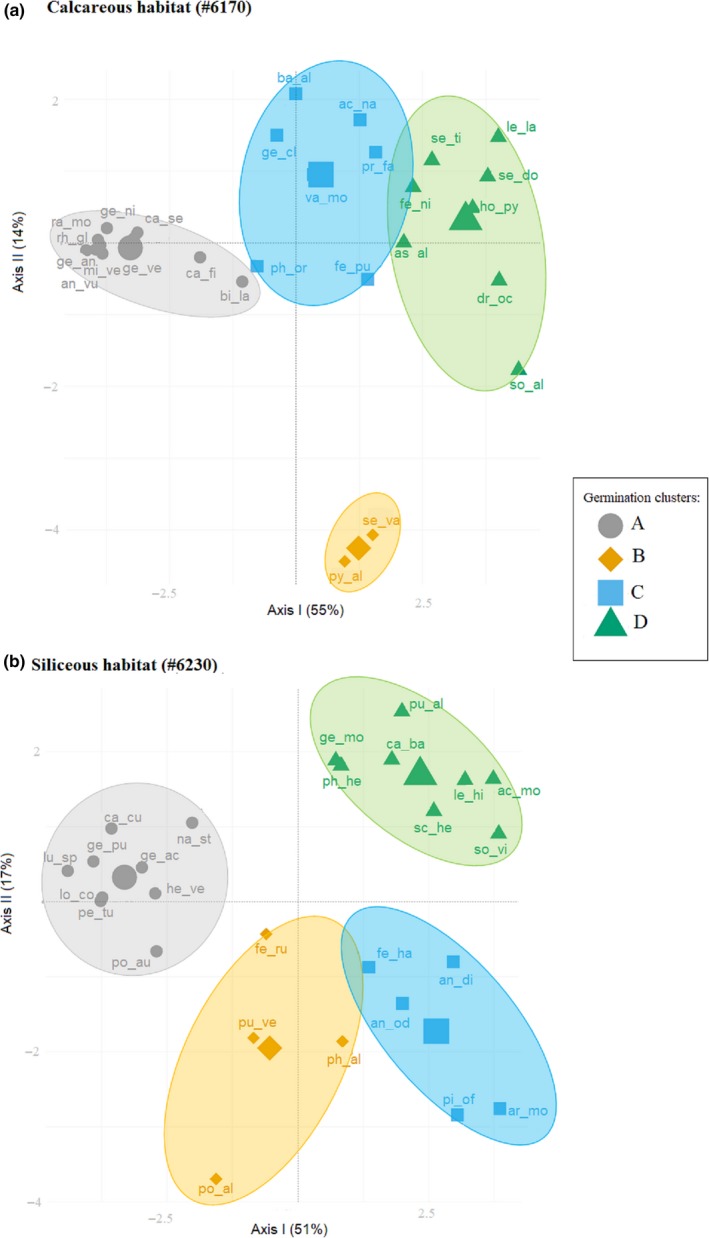
Principal component analysis (PCA) representing the main two axes of variation on the germination patterns. Each spot represents a species indicated by the two first letters in the genus and name. The collections are clustered into four main groups according to their germination strategy. Confidence ellipses represent 0.80 intervals of confidence around the species per cluster (a) Calcareous bedrock. (b) Siliceous bedrock

Moreover, in the calcareous habitat, the cluster analysis (Figure 1a) revealed four major clusters (i.e., A, B, C, and D; Table 2). “Cluster A” represents the lower germinators (i.e., 10 species). “Cluster B” portrays species whose germination decreased after cold stratification, while it was high and rapid on fresh seeds (i.e., two species). “Cluster C” (i.e., seven species) includes the species with slow germination, occurring mostly after the cold stratification. Finally, “Cluster D” (i.e., eight species) represents species with high and rapid germination capabilities, mostly showing only low FGP immediately after dispersal at the low incubation temperature (15/5°C) and/or during the cold stratification (Table 2).

**Table 2:** Germination clusters (A, B, C, and D) and species belonging to each them divided by habitat provenance

Clusters	Species		Germination response
	Siliceous	Calcareous	
A	*Carex curvula, Gentiana acaulis**, Gentiana punctata,** Helictochloa versicolor, Lotus corniculatus, Luzula spicata, Nardus stricta, Pedicularis tuberosa, Potentilla aurea*	*Anthyllis vulneraria, Biscutella laevigata, Carex firma, Carex sempervirens, Gentiana nivalis, Gentiana verna**, Gentianella anisodonta**, Minuartia verna, Rhinanthus glacialis, Ranunculus montanus*	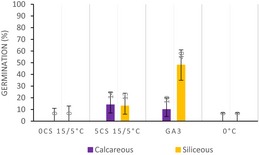
B	*Festuca nigrescens, Phleum raethicum, **Poa alpina**, Pulsatilla vernalis*	*Polygala alpina, **Sesleria caerulea***	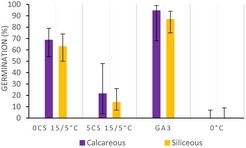
C	*Antennaria dioica, Anthoxanthum odoratum, **Arnica montana**, Festuca halleri, Pilosella officinarum*	*Achillea nana, Bartsia alpina, Festuca pumila, **Gentiana clusii,** Phyteuma orbiculare, Primula farinosa, Valeriana montana*	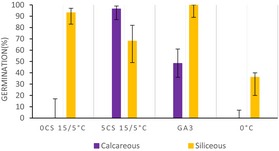
D	***Achillea moschata**, Campanula barbata, Geum montanum, Leontodon hispidus, Pulsatilla alpina**,** Scorzoneroides helvetica, Phyteuma hemisphaericum, Solidago virgaurea*	*Aster alpinus, Dryas octopetala, Festuca nigricans, Horminum pyrenaicum, Leontopodium alpinum, Senecio doronicum, **Serratula tinctoria**, Soldanella alpina*	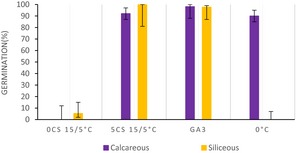

The germination response was summarized using barplots representing FGP (Final germination percentage) scored in some of the conditions tested of one representative specie (in bold) from each germination bedrock, species inhabiting in calcareous habitats in purple and siliceous species’ in yellow.

0 CS 15/5°C. FGP scored of seeds sown after 0 months of cold stratification (or fresh seeds) at 15/5°C incubation temperature.

5 CS 15/5°C. FGP scored of seeds sown after 5 months of cold stratification at 15/5°C incubation temperature.

GA3°C. FGP scored of seeds sown with 250 mg/L of gibberellic acid at 25/15°C incubation temperature.

0°C. FGP scored of seeds sown at 0°C and dark conditions during 5 months.

Overall, the cluster analysis (Figure 1b) displayed a different position of the germination groups and a higher within‐group variability in the siliceous habitat compared to that of the calcareous habitat. Hence, in siliceous clusters species germination responses are more diverse than in the calcareous clusters. Particularly, main differences were observed in “Cluster A” describing again the lower germinators, but also some others that germinated only with GA_3_ (i.e., nine species) and in “Cluster C” (i.e., five species) describing species with high and rapid germination under all conditions (not present in the calcareous habitat), including the emergence during cold stratification. Finally, the species’ categories in clusters B (i.e., five species) and D (i.e., nine species) were similar between the habitats (Table 2).

### 3.2 Dormancy class and level

**Figure 2:**
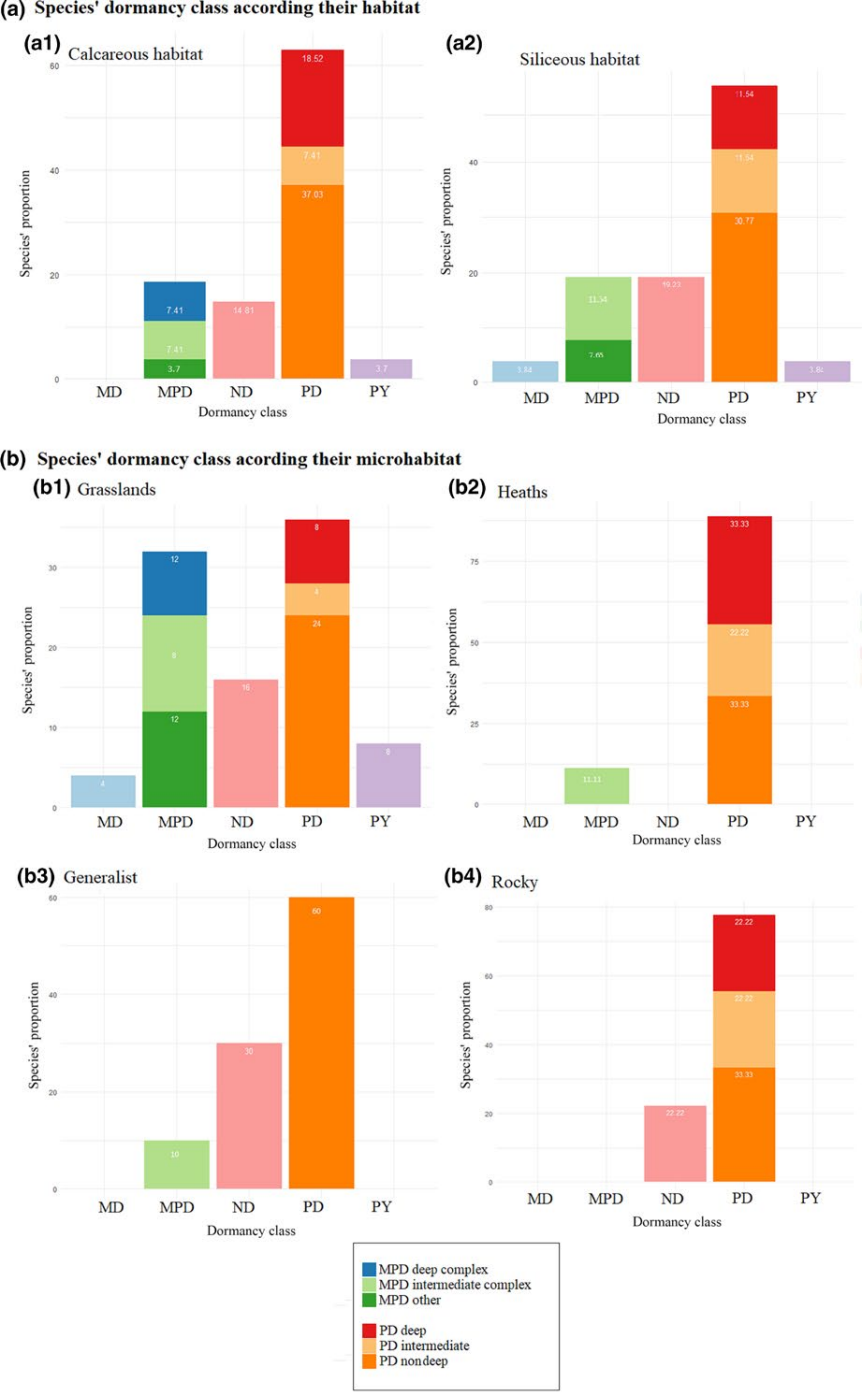
Proportion of dormancy class and level of the target species. MD, morphological dormancy; MPD, morphophysiological dormancy; PY, physical dormancy; PD, physiological dormancy; ND, not dormant within (a) each habitat (from right to left, calcareous and siliceous bedrocks) (b) each microhabitat (from right to left and from top to down: heaths, rocky, generalist, grassland)

Overall, most of the species produced dormant seeds at dispersal (see Table S3b and Figure 2), as only nine species (<20% of those tested) were found to have predominantly nondormant seeds. The most representative dormancy class was PD (32 of 53), and out of those, the non‐deep level was the most abundant (19 of 32), followed by deep (seven of 32) and the remaining six species were classified in the intermediate level. Of eleven species that had undifferentiated or underdeveloped embryos at dispersal, only one (*Pulsatilla vernalis*) was considered to have MD, while the others showed MPD; five species (*Pulsatilla alpina*,* Gentiana clusii*,* Gentiana acaulis*,* Gentiana verna*, and *Campanula barbata*) were classified under the intermediate complex MPD and two species under deep complex type (*Gentiana nivalis* and *Bartsia alpina*). Further studies are needed to classify the remaining three species (i.e., *Gentianella anisodonta*,* Pedicularis tuberosa*, and *Gentiana punctata*) to a MPD level. Finally, only two species (*Lotus corniculatus* and *Anthyllis vulneraria*) were found to be physically dormant with an impermeable seed coat (see Table S3b).

Dormancy was compared between both habitats and microhabitats (Figure 2). At habitat level, no differences were detected in the proportion of species with dormancy class and level. However, differences were found between microhabitat, with species occurring on heaths producing only dormant seeds (c. 89 % PD and 11% MPD), followed by grasslands showing 84% of species with dormant seeds (36% PD; 32% MPD, 8% PY, and 4% MD), rocky with c. 77% of species showing PD and the generalist (60% PD and 10% MPD). Additionally, generalist species showed only the lowest intensity of dormancy (i.e., non‐deep PD), while in the other microhabitats, there was always a given degree of more intense dormancy state, either deep/intermediate‐PD or deep/intermediate/complex‐MPD.

### 3.3 Phylogenetic comparative analysis

**Figure 3:**
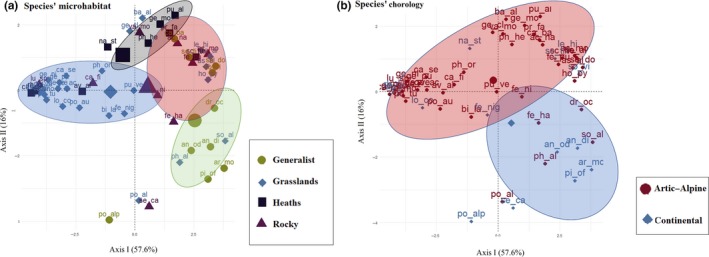
Principal component analysis (PCA) representing the main two axes of variation on the germination patterns (‘germinability’ and ‘dormancy’). Each spot represents a species indicated by the two first letters in the genus name and the specific epithet. The species are colored according to (a) species’ microhabitat occurrence, (b) species’ chorology

Germination traits were summarized using a PCA (Figure 3), in which the two‐principal axis explained 73.65% of the variance (see Table S4). Axis I (representing 57.6% of the variance) corresponded to the ability of germination and is referred to here as “germinability.” It was positively correlated with all the FGPs and negatively with the *T*_50_, and it was mostly explained by the 3 and 5 CS, treatments across both germination test temperatures (see Table S4). Hence, the species with slow and low germinations were separated from the species with fast and quick germinations; for example, *C. curvula* occupies the left part of Axis I, while *A. montana* is on the right. Axis II, explaining 16% of the variance, was negatively correlated with the FGP of 0 CS and positively with *T*_50_ of 0 CS (see Table S4). Hence, species which had the ability to germinate immediately following dispersal, such as *Pilosella officinarum*, appear on the down part of the plot*,* whereas species which did not germinate immediately following dispersal, such as *G. clusii*, appear in the top part of the plot. Therefore, Axis II is consider here as “germinability after dispersal.”

The PGLS was fitted using both Axis I and Axis II, and the best model was selected using the AIC criteria. For this reason, when the AIC decrease and no significant differences between models were detected and also levels’ intercept were similar, the levels were grouped together, following Crawley (2013). So, Chorology (arctic–alpine [including arctic–alpine, alpine, and South‐Europe mountain origin chorotypes] and continental [including Eurosiberian, Euroasiatic, and Europe chorotypes]) and Microhabitats for Axis I (Grasslands‐Heath [including species inhabiting grasslands and heaths], Rocky, and Generalist).

The final models demonstrate that species Axis I scores significantly differ based on species’ microhabitat occurrence (Table 3), the Axis II scores as a function of the chorology and microhabitats (Table 3). The phylogenetic signal (Pagel's λ) associated with the regression residuals was close to 0.6 for Axis I (or germinability) (Table 3), indicating moderate phylogenetic dependence in the relationship. λ was 0.34 for Axis II (Table 3), showing more phylogenetic independence among observations than the previous one. Species with high Axis I scores (see Table S1) belong mostly to the generalist (Figure 3a). In contrast, species with low Axis I scores occur mostly in grasslands. The species with high Axis II scores (see Table S1) mostly occurred on heath and have an arctic–alpine chorotype (Figure 3b). Meanwhile, the species with low Axis II scores are mostly generalists and have a continental chorotype.

**Table 3:** Phylogenetic least squared correlations between germination traits and ecological traits

Models	Factor	Level	Mean	*SE*	*p* Value
Germinability ~ microhabitat	**Microhabitat**	Generalist	1.196a	1.09	
λ = 0.66		Grassland‐Heaths	−0.850b	0.87	
AIC = 242.85		Rocky	0.403ab	1.11	**0.05**
Dormancy ~ microhabitat + Chorology	**Microhabitat**	Generalist	−0.903a	0.52	
		Heath	0.964b	0.48	
		Grasslands	−0.377a	0.37	
λ = −0.34		Rocky	0.039ab	0.48	**0.001**
AIC = 181.22	**Chorology**	Continental	−0.878a	0.47	
		Arctic–alpine	0.074b	0.35	**0.039**

Significant (*p* > .05) values in bold character and differences among levels of a factor indicate by different letters.

### 3.4 Seed weight

Overall, species’ weight differed greatly among species (Table S2). The magnitude of seed weight ranged from 0.00132 g (i.e., *G. nivalis*) to 0.29 g (i.e., *L. corniculatus*), with a mean of 0.0578 g per 50 seeds.

The individual lineal models assessing lineal relationships between seed weight and the two‐principal axis (i.e., Axis I and Axis II) and the log‐transformed FGP resulted in weak relationships (see Table S5). No relations between mean seed weight and habitat or microhabitats were detected (see Table S5).

## 4 DISCUSSION

### 4.1 Habitat‐related seed germination traits

Our results show that habitat provenience has a significant effect on FGP and on the germination strategies (i.e., clusters) in each bedrock, indicating the presence of habitat‐related regenerative strategies. In particular, species from “Cluster C” in the calcareous grasslands showed a slow germination and mostly after cold stratification (i.e., see middle‐upper part of Figure 1a). Consequently, these species would germinate mostly in spring when there is a lower risk of heat stress, with the remaining ungerminated seeds forming a persistent soil seed bank. Interestingly, topsoil desiccation and temporary stresses such as wind erosion and hyperthermia in summer (Gigon, 1942) are common in calcareous bedrocks (Kammer & Mohl, 2016) and may strongly affect seedling establishment (Marcante, Erschbamer, Buchner, & Neuner, 2014). This may explain why species from the calcareous grasslands displayed a significantly higher FGP during the cold stratification period, when water availability is high. As a result, temporally spread winter emergence would allow some seedlings to reach the summer drought period at a developed stage; for example, young plants would have a more deeper root system and deeper soils layers rarely dry up (Körner, 2003). Conversely, in siliceous grasslands, characterized by lower risk of drought, the germination strategy found in “Cluster C” showed no restrictions, germinating to high levels in all conditions tested (i.e., see right‐down part of Figure 1b). These results indicate that species from siliceous soils are able to stagger their germination throughout the whole year depending on the environmental conditions. For instance, if summer temperatures are warm enough, a proportion of the population would be able to germinate, though, in cold summer years, the germination can be postponed after the snowmelt, with a proportion of seeds germinating under the snow. This germination plasticity may potentially increase their regeneration capacity, though depleting the soil seed bank. Moreover, soil water availability during seed development and maturation is also known to have large influence in seed germination requirements, via maternal effect (Lu, Tan, Baskin, & Baskin, 2016). Therefore, the possibility that habitat dormancy differences may be related to the environmental heterogeneity of the growing site cannot be ruled out.

Interestingly, “clusters A, B and D” show common germination strategies in both bedrocks, having similar position in the PCA axis (Figure 1). The low germination of the species belonging to “Cluster A” may indicate that cold stratification is not always effective in promoting germination for alpine species or that appropriate cues for germination were not meet (Donohue, Rubio de Casas, Burghardt, Kovach, & Willis, 2010). The low germination of alpine species under laboratory conditions has been suggested as a mechanism to ensure the formation of a persistent soil seed bank (Mondoni et al., 2012; Shimono & Kudo, 2005), as an ecological strategy to the low chance of establishment in these environments (Erschbamer, Niederfriniger Schlag, & Winkler, 2008; Schwienbacher, Marcante, & Erschbamer, 2010), due to summer drought/heat and early autumn/spring frost episodes (Graae et al., 2009; Marcante, Sierra‐Almeida, Spindelböck, Erschbamer, & Neuner, 2012; Marcante et al., 2014).

The strategy summarized for “Cluster B” is represented by species with nondormant seeds, which germinate after dispersal, in late summer or autumn. These species probably remain in the soil surface if they required light for germination or they are species able to germinate under dark conditions. In addition, for some species (i.e., *Sesleria varia*,* Polygala alpina*,* Poa alpina*,* P. vernalis*, and *Festuca rubra*) belonging to this germination strategy, cold stratification significantly reduces FGP compared with 0 CS, that is, potentially induces deeper dormancy. Despite the fact that autumn germination in alpine habitats has often been considered disadvantageous, recent studies have shown that a high number of autumn‐emerged seedlings could survive winter on glacier forelands (Marcante et al., 2012; Mondoni et al., 2015). Autumn germination may be advantageous for species that produce seeds with short longevity and unlikely to form a persistent seed banks, and represents an ecological advantage that presumably ensures seedlings are well placed to grow quickly when temperatures begin to rise in early spring. Finally, species belonging to “Cluster D” show the typical alpine germination response, postponing germination until late winter or early spring. Overall, seed germination and seedling establishment of alpine plants tends to occur rapidly after snowmelt (Körner, 2003; Schwienbacher et al., 2011), when there is lower risk of frost and temperatures rise fast (Rosbakh & Poschlod, 2015). These conditions are favorable for seedling recruitment as the plants have the entire growing season to reach their optimal size for overwintering (Billings & Mooney, 1968) and early germination has a strong competitive advantage (Grime, 2002).

### 4.2 Phylogenetic comparative analysis

Germination traits (Axis I and Axis II) were weakly correlated with species’ weight and no differences on mean seed weight detected between habitats and microhabitats (see Table S5). Therefore, seed weight did not contribute on explaining local vegetation patterns here, although it is the main regenerative traits used in community ecology publications (Jiménez‐Alfaro et al., 2016). On the other hand, germination traits were strongly correlated with the microhabitat of the species occurrence and with species’ chorotype, highlighting that such factors may importantly contribute to affect plant regeneration strategies. In particular, species inhabiting in heaths showed high Axis II scores, indicating that germinability is high only after dormancy is broken (i.e., after cold stratification), hence that most of these species have dormant seeds. Consistently, all species from heaths were classified as having dormant seeds, with about one third showing deep PD. Moreover, scores levels of Axis II were the lowest in the generalists, as well as the presence and the level of dormancy (60% non‐deep PD; 10% MPD; Figure 2b). Our results also showed differences among Axis I scores, with generalist species having higher scores than those from grasslands, confirming that dormancy state is weak in the former. Accordingly, generalist species showed the highest percentage of ND seeds (30%) and grasslands were among the most dormant, showing 84% of species with dormant seeds, including deep PD and MPD. Following the view of Grubb (1977) and subsequent observations (Huang, Liu, Bradford, Huxman, & Venable, 2016), which highlighted the ecological role of the regeneration niche (including germination traits) in driving plant distribution, our results show that dormancy presence and levels were strongly related to the species occurrence in the microhabitats. This is an interesting and novel observation, which help explaining the large diversity of germination and dormancy responses in alpine environments and add novel insights to their possible functional role as drivers of species distribution in alpine environment (sensu Jiménez‐Alfaro et al., 2016).

As dormancy is a mechanism of plants to synchronize germination with a suitable moment for seedling recruitment (Baskin & Baskin, 2014) and sets the context for plant development (Donohue et al., 2010), the different germination behaviors found here indicate that recruitments from seeds follow different patterns in each microhabitat. However, such possibility cannot be fully understood without a detailed investigation of the microclimate in each habitat, population density in each microhabitat, and/or in situ germination evidences. Therefore, any explanation of the possible environmental cue affecting the major germination strategy in each microhabitat is purely speculative, though worthy of comments. For example, plants inhabiting wind‐exposed and steep places such as often happen in sedge heaths (Nagy & Grabherr, 2009) would expose seedlings to high risk of frost and desiccation stress in the case of autumn emergence. Consequently, plants inhabiting in heaths produce seeds capable of maintaining deep dormancy until winter has passed. On the other hand, in snowbeds, where some generalists occur, thermal buffering under constant snow cover protects any emergence seedlings during winter (Schaberg, Hennon, D'amore, & Hawley, 2008), which may explain why dormancy was weaker in this microhabitat. Again, the high germination capacity of generalist species may increase the likelihood of seedling recruitment in different microhabitats. Indeed, with wider germination conditions may be exposed to more spatial and temporal establishment opportunities and occupied different types of environments (Thompson & Ceriani, 2003). Meanwhile, the narrower and lower germination conditions showed by most of the species occurring in grasslands may indicate that the appropriate germination cuing was lacking or that longer soil seed banks are expected in these species. Seeds of alpine plants have been shown to form persistent soil seed banks (Schwienbacher et al., 2010), providing an ecological advantage by avoiding unfavorable environmental conditions for seedling establishment (Ooi, 2012). It should be clear that while we used the term “species” here to highlight species‐specific germination strategy linked the two habitats, any interpretation in an evolutionary context must be considered limited to the individuals and their populations here studied.

Finally, in our study arctic/alpine chorotypes (arctic–alpine, alpine, and south‐Europe‐montane) gave higher dormancy scores (Axis II) than continental species. This difference supports the hypothesis that seed dormancy is partly influenced by the natural life history of plants (Schaal & Leverich, 1981) and, hence, by the environmental conditions experienced in the past. In this case, selection pressure on montane areas acts to prevent germination after dispersal (e.g., through a higher dormancy state) and decrease the probability of encountering subsequent unfavorable growth conditions (Probert, 2000), which may explain our findings.

## CONFLICT OF INTEREST

None declared.

## AUTHOR CONTRIBUTIONS

Andrea Mondoni, Hugh Pritchard, Simone Orsenigo, Graziano Rossi, and Maria Tudela‐Isanta conceived the ideas and designed methodology; Maria Tudela‐Isanta, Graziano Rossi, Simone Orsenigo, and Malaka Wijayasinghe collected the data; Maria Tudela‐Isanta and Eduardo Fernandez‐Pascual analyzed the data; Andrea Mondoni and Maria Tudela‐Isanta led the writing of the manuscript. All authors contributed critically to the drafts and gave final approval for publication.
