# Supplementary material for: Habitat‐related seed germination traits in alpine habitats
Source: Ecol Evol. 2017 Nov 24;8(1):150–61. doi: 10.1002/ece3.3539 (PMC5756861; doi:10.1002/ece3.3539)
Supplement: Supplementary file 1 [file ECE3-8-150-s001.docx]

**Table S1*.*** Germination traits and species microhabitat and chorology for the target species.

| Species | Species characteristics | | Germination traits | |
| --- | --- | --- | --- | --- |
|  |  | |  | |
|  | Microhabitat^a^ | Chorology^a^ | Axis I | Axis II |
| *Achillea moschata* Wulfen | Rocky | Artic-Alpine | 3.081 | 1.039 |
| *Achillea nana* L. | Rocky | Artic-Alpine | 2.260 | 1.273 |
| *Antennaria dioica* (L.) Gaertn. | Generalist | Continental | 3.259 | -1.812 |
| *Anthoxanthum odoratum* L. | Generalist | Continental | 2.603 | -1.935 |
| *Anthyllis vulneraria* L. | Grasslands | Continental | -3.899 | -0.071 |
| *Arnica montana* L. | Generalist | Continental | 3.710 | -2.445 |
| *Aster alpinus* L. | Grasslands | Artic-Alpine | 2.985 | 0.676 |
| *Bartsia alpina* L. | Grasslands | Artic-Alpine | 0.967 | 2.126 |
| *Biscutella laevigata* L. | Grasslands | Artic-Alpine | -1.545 | -0.810 |
| *Campanula barbata* L. | Generalist | Artic-Alpine | 1.712 | 1.625 |
| *Carex curvula* All. | Heaths | Artic-Alpine | -4.192 | -0.072 |
| *Carex firma* Host | Rocky | Artic-Alpine | -2.215 | 0.282 |
| *Carex sempervirens* Vill. | Grasslands | Artic-Alpine | -2.884 | 0.405 |
| *Dryas octopetala* L. | Generalist | Artic-Alpine | 3.315 | -0.614 |
| *Festuca halleri* All. | Rocky | Artic-Alpine | 1.844 | -0.999 |
| *Festuca nigricans* (Hack.) K.Richt. | Rocky | Artic-Alpine | 2.518 | 0.820 |
| *Festuca pumila* Chaix. | Rocky | Artic-Alpine | 1.241 | -0.149 |
| *Festuca nigrescens Lam.* | Grasslands | Continental | -0.161 | -0.639 |
| *Gentiana acaulis* L. | Grasslands | Artic-Alpine | -3.430 | 0.267 |
| *Gentiana clusii* E.P. Perrier & Songeon. | Grasslands | Artic-Alpine | 0.241 | 1.876 |
| *Gentiana nivalis* L. | Grasslands | Artic-Alpine | -3.489 | 0.279 |
| *Gentiana punctata* L. | Grasslands | Artic-Alpine | -3.368 | 0.320 |
| *Gentiana verna* L. | Grasslands | Artic-Alpine | -3.691 | 0.243 |
| *Gentianella anisodonta* (Borbás) Á. & D.Löve | Grasslands | Artic-Alpine | -4.086 | -0.017 |
| *Geum montanum* L. | Heaths | Artic-Alpine | 1.674 | 1.901 |
| *Helictochloa versicolor* (Vill.) Romero Zarco | Heaths | Artic-Alpine | -2.565 | 0.026 |
| *Horminum pyrenaicum* L. | Grasslands | Artic-Alpine | 3.101 | 0.253 |
| *Leontodon hispidus* L. | Grasslands | Continental | 2.857 | 1.159 |
| *Leontopodium alpinum* Cass. | Grasslands | Artic-Alpine | 3.152 | 0.972 |
| *Lotus corniculatus* L. | Grasslands | Continental | -3.129 | -0.478 |
| *Luzula spicata* (L.) DC. | Rocky | Artic-Alpine | -3.859 | 0.160 |
| *Minuartia verna* (L.) Hiern subsp. *verna* | Grasslands | Artic-Alpine | -3.768 | 0.016 |
| *Nardus stricta* L. | Heaths | Continental | -1.217 | 1.159 |
| *Pedicularis tuberosa* L. | Grasslands | Artic-Alpine | -4.177 | -0.065 |
| *Phleum rhaeticum* (Humphries) Rauschert | Grasslands | Artic-Alpine | 1.310 | -2.395 |
| *Phyteuma hemisphaericum* L. | Heaths | Artic-Alpine | 0.601 | 1.604 |
| *Phyteuma orbiculare* L. | Grasslands | Artic-Alpine | -1.315 | 0.932 |
| *Pilosella officinarum* Vaill. | Generalist | Continental | 3.237 | -2.828 |
| *Poa alpina* L. | Generalist | Continental | -1.655 | -3.920 |
| *Polygala alpina* (DC.) Steud. | Grasslands | Artic-Alpine | 0.301 | -3.229 |
| *Potentilla aurea* L. | Grasslands | Artic-Alpine | -2.734 | -0.455 |
| *Primula farinosa* L. | Heaths | Artic-Alpine | 1.511 | 1.818 |
| *Pulsatilla alpina* L. | Heaths | Artic-Alpine | 1.749 | 2.371 |
| *Pulsatilla vernalis* (L.) Mill. | Grasslands | Artic-Alpine | -0.200 | -0.259 |
| *Ranunculus montanum* Willd. | Grasslands | Artic-Alpine | -3.720 | -0.132 |
| *Rhinanthus glacialis* Personnat | Heaths | Continental | -4.014 | 0.009 |
| *Scorzoneroides helvetica* (Mérat) Holub | Heaths | Artic-Alpine | 2.607 | 0.982 |
| *Senecio doronicum* (L.) L. | Generalist | Artic-Alpine | 3.481 | 0.675 |
| *Serratula tinctoria* L. | Generalist | Continental | 1.653 | 0.846 |
| *Sesleria caerulea* (L.) Ard. | Rocky | Continental | 0.627 | -3.409 |
| *Soldanella alpina* L. | Grasslands | Artic-Alpine | 3.755 | -1.635 |
| *Solidago virgaurea* L. | Generalist | Continental | 3.288 | 0.450 |
| *Valeriana montana* L. | Rocky | Artic-Alpine | 0.669 | 1.795 |

^a^ All based from environmental code and world distribution by Aeschimann et al. 2004

**Table S2**. Species germination outputs (including the mean FGP of each specie and the T50) for each treatment.

|  |  |  | 0°C | 15/5°C | | | | | | 25/15°C | | | | | | | |
| --- | --- | --- | --- | --- | --- | --- | --- | --- | --- | --- | --- | --- | --- | --- | --- | --- | --- |
|  |  |  |  | 0CS | | 3CS | | 5CS | | 0CS | | 3CS | | 5CS | | GA3 | |
| Species | acronym | weight  (g) | FGP  (%) | FGP  (%) | T_50_  (days) | FGP  (%) | T_50_  (days) | FGP  (%) | T_50_  (days) | FGP  (%) | T_50_  (days) | FGP  (%) | T_50_  (days) | FGP  (%) | T_50_  (days) | FGP  (%) | T_50_  (days) |
| *Achillea moschata* Wulfen | ac_mo | 0.009 | 0 | 5.3 | NA | 100 | 93.37 | 100 | 152.77 | 86.3 | 9.58 | 100 | 95.10 | 100 | 150.99 | 97.7 | 3.61 |
| *Achillea nana* L. | ac_na | 0.013 | 0 | 3.4 | NA | 77.8 | 99.08 | 84.8 | 152.96 | 37.5 | 46.25 | 91.7 | 94.94 | 94.4 | 151.07 | 37.4 | NA |
| *Antennaria dioica* (L.) Gaertn. | an_di | 0.003 | 0 | 64.4 | 14.95 | 100 | 95.17 | 89.5 | 152.60 | 81.5 | 4.20 | 82.5 | 94.26 | 71.7 | 157.02 | 85.0 | 3.69 |
| *Anthoxanthum odoratum* L. | an_od | 0.022 | 5.5 | 56.6 | 19.16 | 72.6 | 100.91 | 79.9 | 158.03 | 81.3 | 3.21 | 62.7 | 102.25 | 60.1 | 159.72 | 64.5 | 15.70 |
| *Anthyllis vulneraria* L. | an_vu | 0.208 | 41.0 | 1.6 | NA | 5.3 | NA | 8.6 | NA | 9.0 | NA | 6.7 | NA | 3.8 | NA | 6.8 | NA |
| *Arnica montana* L. | ar_mo | 0.076 | 36.8 | 93.0 | 12.35 | 100 | 93.58 | 68.0 | 151.19 | 90.0 | 5.79 | 96.5 | 90.97 | 90.0 | 151.05 | 100 | 1.06 |
| *Aster alpinus* L. | as_al | 0.315 | 1.0 | 18.1 | NA | 95.8 | 96.71 | 92.4 | 154.22 | 86.1 | 7.05 | 92.4 | 94.22 | 100 | 152.84 | 100 | 6.82 |
| *Bartsia alpina* L. | av_al | 0.013 | 2.0 | 0.0 | NA | 94.1 | NA | 90.4 | NA | 0.0 | NA | 56.7 | NA | 50.9 | NA | 20.6 | 11.40 |
| *Biscutella laevigata* L. | ba_al | 0.284 | 0 | 4.6 | NA | 5.8 | 97.35 | 13.1 | 160.17 | 73.7 | NA | 58.2 | 96.07 | 31.6 | 152.06 | 73.9 | NA |
| *Campanula barbata* L. | bi_la | 0.003 | 2.5 | 12.0 | NA | 80.0 | NA | 63.3 | NA | 40.0 | 15.69 | 100 | 105.82 | 93.3 | NA | 98.3 | 15.54 |
| *Carex curvula* All. | ca_ba | 0.111 | 0 | 0.0 | NA | 0.00 | 103.72 | 0.0 | 169.36 | 0.0 | NA | 0.0 | 91.06 | 0.0 | 156.86 | 0.0 | 5.60 |
| *Carex firma* Host | ca_cu | 0.032 | 0 | 2.2 | NA | 35.6 | NA | 49.1 | NA | 50.0 | NA | 42.2 | NA | 49.4 | NA | 79.1 | NA |
| *Carex sempervirens* Vill. | ca_fi | 0.100 | 0 | 1.6 | NA | 16.6 | NA | 20.0 | NA | 1.6 | NA | 10.0 | NA | 23.7 | NA | 23.3 | 14.27 |
| *Dryas octopetala* L. | ca_se | 0.043 | 0 | 7.1 | NA | 100 | NA | 100 | NA | 100 | NA | 91.7 | NA | 84.4 | NA | 100 | NA |
| *Festuca halleri* All. | dr_oc | 0.017 | 0 | 10.1 | 70.25 | 64.1 | 91.06 | 57.4 | 151.06 | 62.5 | 3.58 | 65.0 | 93.82 | 53.7 | 151.13 | 69.4 | 5.72 |
| *Festuca nigricans* (Hack.) K.Richt. | fe_ha | 0.048 | 6.6 | 13.6 | 47.73 | 83.3 | 97.00 | 55.6 | 163.77 | 58.6 | 22.69 | 91.1 | 99.37 | 100 | 161.59 | 88.9 | 8.48 |
| *Festuca pumila* Chaix. | fe_ni | 0.030 | 70.8 | 28.7 | NA | 55.0 | 100.59 | 61.1 | 161.55 | 69.2 | 17.44 | 39.2 | NA | 100 | 151.06 | 62.0 | 16.65 |
| *Festuca nigrescens Lam.* | fe_nig | 0.041 | 0.18 | 22.4 | NA | 32.8 | NA | 50.0 | 156.80 | 70.2 | 14.13 | 48.3 | NA | 50.0 | 157.30 | 63.0 | 7.66 |
| *Gentiana acaulis* L. | fe_pu | 0.017 | 0 | 3.3 | NA | 26.4 | 96.58 | 41.7 | 151.49 | 1.7 | 27.51 | 10.0 | 95.12 | 0.0 | 156.52 | 10.0 | 8.55 |
| *Gentiana clusii* E.P. Perrier & Songeon. | ge_ac | 0.012 | 0 | 0.0 | NA | 90.3 | NA | 96.4 | NA | 0.0 | NA | 39.1 | NA | 52.4 | NA | 48.3 | 12.56 |
| *Gentiana nivalis* L. | ge_an | 0.001 | 0 | 0.0 | NA | 35.4 | NA | 20.4 | NA | 0.0 | NA | 20.5 | NA | 0.0 | NA | 0.0 | NA |
| *Gentiana punctata* L. | ge_cl | 0.100 | 0 | 0.0 | NA | 25.7 | 107.24 | 13.0 | 164.22 | 0.0 | NA | 32.2 | NA | 15.1 | 166.83 | 48.0 | NA |
| *Gentiana verna* L. | ge_mo | 0.002 | 0 | 0.0 | NA | 0.0 | 94.53 | 29.5 | 151.06 | 0.0 | NA | 0.0 | 94.82 | 24.5 | 151.05 | 84.0 | NA |
| *Gentianella anisodonta* (Borbás) Á. & D.Löve | ge_ni | 0.007 | 11.6 | 0.0 | NA | 1.9 | NA | 14.0 | NA | 0.0 | NA | 1.7 | NA | 7.2 | NA | 10.0 | NA |
| *Geum montanum* L. | ge_pu | 0.043 | 8.3 | 16.2 | NA | 97.6 | NA | 84.9 | NA | 11.9 | NA | 83.9 | NA | 81.5 | NA | 24.2 | NA |
| *Helictochloa versicolor* (Vill.) Romero Zarco | ge_ve | 0.085 | 10.0 | 12.1 | NA | 26.3 | NA | 34.3 | NA | 34.9 | NA | 45.5 | NA | 35.5 | NA | 66.4 | 8.54 |
| *Horminum pyrenaicum* L. | ho_py | 0.035 | 0 | 31.2 | NA | 100 | 98.82 | 80.6 | 162.47 | 96.5 | 4.09 | 100 | 91.06 | 96.3 | 151.93 | 94.1 | 1.93 |
| *Leontodon hispidus* L. | le_al | 0.070 | 0 | 7.7 | NA | 100 | 96.76 | 92.7 | 157.05 | 61.8 | 0.67 | 100 | 91.06 | 100 | 150.97 | 91.2 | 6.29 |
| *Leontopodium alpinum* Cass. | le_hi | 0.006 | 0 | 3.3 | NA | 100 | 95.00 | 100 | 151.40 | 98.3 | 9.05 | 100 | 94.07 | 100 | 151.06 | 100 | 2.04 |
| *Lotus corniculatus* L. | lo_co | 0.290 | 0 | 19.3 | NA | 21.7 | NA | 18.4 | NA | 37.5 | NA | 28.3 | NA | 5.0 | NA | 31.4 | NA |
| *Luzula spicata* (L.) DC. | lu_sp | 0.013 | 0 | 0.0 | NA | 0.0 | NA | 31.7 | NA | 0.0 | NA | 0.0 | NA | 3.3 | NA | 0.0 | NA |
| *Minuartia verna* (L.) Hiern subsp. *verna* | mi_ve | 0.007 | 7.5 | 1.8 | NA | 9.2 | NA | 11.0 | NA | 6.8 | NA | 13.3 | NA | 3.3 | NA | 10.0 | NA |
| *Nardus stricta* L. | na_st | 0.019 | 0 | 0.0 | NA | 33.0 | NA | 40.4 | NA | 3.9 | NA | 52.8 | 110.97 | 64.2 | 159.00 | 30.6 | NA |
| *Pedicularis tuberosa* L. | pe_tu | 0.050 | 0 | 0.0 | NA | 1.7 | NA | 0.0 | NA | 0.0 | NA | 0.0 | NA | 0.0 | NA | 85.0 | 17.72 |
| *Phleum rhaeticum* (Humphries) Rauschert. | ph_al | 0.013 | 18.0 | 56.4 | 21.22 | 64.8 | NA | 50.8 | NA | 80.3 | 6.77 | 74.0 | 94.20 | 80.0 | 152.25 | 87.5 | 6.66 |
| *Phyteuma hemisphaericum* L. | ph_he | 0.013 | 2.5 | 14.2 | NA | 100 | 96.16 | 84.6 | 157.42 | 8.6 | NA | 75.9 | 93.33 | 40.5 | NA | 100 | 3.70 |
| *Phyteuma orbiculare* L. | ph_or | 0.007 | 52.5 | 6.8 | NA | 65.4 | 105.61 | 30.6 | 192.00 | 1.8 | NA | 31.2 | NA | 24.2 | NA | 100 | 7.91 |
| *Pilosella officinarum* Vaill. | pi_of | 0.007 | 20.0 | 1.0 | 6.76 | 90.3 | 95.57 | 59.4 | 156.05 | 86.7 | 2.34 | 100 | 91.06 | 55.6 | 151.59 | 72.7 | 1.26 |
| *Poa alpina* L. | po_al | 0.028 | 7.0 | 62.9 | 14.20 | 18.3 | 120.46 | 13.8 | 192.92 | 88.9 | 13.60 | 21.7 | NA | 16.9 | NA | 86.8 | 7.92 |
| *Polygala alpina* (DC.) Steud. | po_alp | NA | 6.0 | 74.4 | 13.74 | 50.0 | NA | 30.4 | NA | 71.1 | 5.61 | 36.1 | NA | 35.7 | NA | 78.1 | 4.25 |
| *Potentilla aurea* L. | po_au | 0.015 | 0 | 1.7 | NA | 13.4 | NA | 24.0 | NA | 13.5 | 41.99 | 30.1 | NA | 25.0 | NA | 72.6 | 19.26 |
| *Primula farinosa* L. | pr_fa | 0.003 | 0 | 11.7 | NA | 75.9 | 101.85 | 83.4 | 161.59 | 21.2 | NA | 91.7 | 94.59 | 76.7 | 153.38 | 98.2 | 6.31 |
| *Pulsatilla alpina* L. | pu_al | 0.166 | 40.0 | 0.0 | NA | 100 | 100.69 | 83.3 | 151.04 | 6.7 | NA | 100 | 97.29 | 75.6 | 155.26 | 100 | 13.63 |
| *Pulsatilla vernalis* (L.) Mill. | pu_ve | 0.075 | 0 | 0.0 | NA | 27.3 | NA | 40.7 | NA | 85.0 | 17.63 | 56.8 | 111.54 | 60.2 | 166.87 | 33.3 | NA |
| *Ranunculus montanum* Willd. | ra_mo | 0.031 | 6.6 | 5.0 | NA | 8.6 | NA | 7.9 | NA | 13.7 | NA | 15.3 | NA | 3.3 | NA | 8.6 | NA |
| *Rhinanthus glacialis* Personnat | rh_gl | 0.124 | 54.2 | 0.0 | NA | 0.0 | NA | 0.0 | NA | 0.0 | NA | 0.0 | NA | 0.0 | NA | 0.0 | NA |
| *Scorzoneroides helvetica* (Mérat) Holub | sc_he | 0.080 | 0 | 5.3 | NA | 88.3 | 94.05 | 79.9 | 154.37 | 69.6 | 2.72 | 93.3 | 91.57 | 100 | 151.06 | 95.0 | 1.78 |
| *Senecio doronicum* (L.) L. | se_ca | 0.142 | 90.0 | 25.1 | 13.10 | 100 | 103.81 | 100 | 154.40 | 92.6 | 14.13 | 92.6 | NA | 100 | 151.06 | 89.4 | 2.83 |
| *Serratula tinctoria* L. | se_do | 0.079 | 0 | 0.0 | NA | 98.1 | 91.06 | 92.1 | 150.97 | 76.5 | 9.23 | 100 | 99.23 | 100 | 150.97 | 98.1 | 0.97 |
| *Sesleria caerulea* (L.) Ard. | se_ti | 0.008 | 8.3 | 68.6 | NA | 63.4 | 94.48 | 21.4 | NA | 97.6 | 6.49 | 11.1 | 91.06 | 11.1 | NA | 94.4 | 11.81 |
| *Soldanella alpina* L. | so_al | 0.012 | 0 | 57.9 | 28.29 | 98.0 | 103.67 | 95.5 | 160.40 | 100 | 12.80 | 96.1 | 99.46 | 100 | 157.39 | 100 | 12.19 |
| *Solidago virgaurea* L. | so_vi | 0.033 | 5.0 | 26.8 | NA | 100 | 93.79 | 100 | 150.88 | 100 | 4.77 | 100 | 95.86 | 95.0 | 155.65 | 93.0 | 5.68 |
| *Valeriana montana* L. | va_mo | 0.043 | 16.0 | 9.0 | NA | 89.1 | 98.28 | 78.9 | 156.88 | 3.0 | NA | 47.1 | NA | 97.8 | 150.98 | 45.3 | NA |

**Table S3a.** Dichotomous key to the 5 class and level of dormancy of the 53 target alpine species interfered from germination tests and literature (e.g. seed coat permeability and embryo type, see table Supplementary material 1b).

1. FGP _0CS_  at 25/15 and 15/5°C is ≥70%........................................................................................**ND**
2. FGP _0CS_  at 25/15 and 15/5°C is ≤70%..........................................................................................**.3**
3. Is seed coat permeable?
   1. Seed coat is not permeable………………………………………….........………………**PY**
   2. Seed coat is permeable………………………………………………….........……………**4**
4. Is embryo fully developed when dispersal?
   1. Embryo is undifferentiated or underdeveloped when dispersal…………………………. **5**
   2. Embryo is fully developed when dispersal ……………………………...………………. **7**
5. Is FGP ≥70%?
   1. Yes……………………………………………………………………………………...**MD**
   2. No………………………………...……………………………………………………….**6**
6. Embryo grows during cold stratification (3 or 5 months)
   1. Gibberellic acid substituted for cold stratification in promoting germination…………………………………….……………**MPD-intermediate complex**
   2. Gibberellic acid did not substitute for cold stratification in promoting germination………………………………….……………………… **MPD- Deep complex**
   3. Gibberellic acid or cold stratification does not promote germination....……**unknow-MPD**
7. Long periods of cold stratification increase significantly FGP.
   1. FGP_0cs_ 25/15°C was high………………………………..…………………... **non-deep PD**
   2. FGP_0cs_ 25/15°C was low and gibberellic acid or short periods (3 months) substituted long period (5CS) of cold stratification in promoting germination...…………...**intermediate PD**
   3. FGP_0cs_ 25/15°c was low and gibberellic acid or short periods (3 months) did not substitute long period (5CS) of cold stratification in promoting germination………………. **deep PD**
   4. FGP was low in all conditions tested…………………………………………...…. **deep PD**

**Table S3b.** Seed dormancy class and level based on the data of germination experiments and the reference species with a literature-based description of endosperm characteristics, embryo type and water impermeability of the seed coat (PWI).

| Species | Family | Embryo type^a^ | Endosperm^b^ | PWI^c^ | Dormancy | |
| --- | --- | --- | --- | --- | --- | --- |
|  |  |  |  |  | Class | Level |
| *Achillea moschata* Wulfen | Asteracea | spatulate fully developed | no endosperm | No | PD | Non deep |
| *Achillea nana* L. | Asteracea | spatulate fully developed | no endosperm | No | PD | Intermediate |
| *Antennaria dioica* (L.) Gaertn. | Asteracea | spatulate fully developed | no endosperm | No | PD | Non deep |
| *Anthoxanthum odoratum* L. | Poaceae | lateral | starchy endosperm | No | ND |  |
| *Anthyllis vulneraria* L. | Fabaceae | bent | fleshy endosperm | Yes | PY |  |
| *Arnica montana* L. | Asteracea | spatulate fully developed | non starchy endosperm | No | PD | Non deep |
| *Aster alpinus* L. | Asteracea | spatulate fully developed | no endosperm | No | PD | Non deep |
| *Bartsia alpina* L. | Orobanchaceae | undifferentiated | fleshy endosperm | No | MPD | Deep complex |
| *Biscutella laevigata* L. | Brassicaceae | n.a | fleshy endosperma | No | ND |  |
| *Campanula barbata* L. | Campanulaceae | linear underdeveloped | fleshy endosperm | No | MPD | Intermediate complex |
| *Carex curvula* All. | Cyperaceae | capitate | starchy endosperm | No | PD | Deep |
| *Carex firma* Host | Cyperaceae | capitate | starchy endosperm | No | PD | Intermediate |
| *Carex sempervirens* Vill. | Cyperaceae | capitate | starchy endosperm | No | PD | Deep |
| *Dryas octopetala* L. | Rosaceae | spatulate fully developed | fleshy endosperm | No | PD | Non deep |
| *Festuca halleri* All. | Poaceae | lateral | starchy endosperm | No | PD | Non deep |
| *Festuca nigricans* (Hack.) K.Richt. | Poaceae | lateral | starchy endosperm | No | PD | Non deep |
| *Festuca pumila* Chaix. | Poaceae | lateral | starchy endosperm | No | ND |  |
| *Festuca nigrescens Lam.* | Poaceae | lateral | starchy endosperm | No | ND |  |
| *Gentiana acaulis* L. | Gentianaceae | linear underdeveloped | fleshy endosperm | No | MPD | Intermediate complex |
| *Gentiana clusii* E.P. Perrier & Songeon. | Gentianaceae | linear underdeveloped | fleshy endosperm | No | MPD | Intermediate complex |
| *Gentiana nivalis* L. | Gentianaceae | linear underdeveloped | fleshy endosperm | No | MPD | Deep complex |
| *Gentiana punctata* L. | Gentianaceae | linear underdeveloped | fleshy endosperm | No | MPD |  |
| *Gentiana verna* L. | Gentianaceae | linear underdeveloped | fleshy endosperm | No | MPD | Intermediate complex |
| *Gentianella anisodonta* (Borbás) Á. & D.Löve | Gentianaceae | linear underdeveloped | fleshy endosperm | No | MPD |  |
| *Geum montanum* L. | Rosaceae | spatulate fully developed | no endosperm | No | PD | Intermediate |
| *Helictochloa versicolor* (Vill.) Romero Zarco | Poaceae | lateral | starchy endosperm | No | PD | Intermediate |
| *Horminum pyrenaicum* L. | Lamiaceae | n.a | n.a | No | PD | Non deep |
| *Leontodon hispidus* L. | Asteracea | spatulate fully developed | no endosperm | No | PD | Non deep |
| *Leontopodium alpinum* Cass. | Asteracea | spatulate fully developed | no endosperm | No | PD | Non deep |
| *Lotus corniculatus* L. | Fabaceae | bent | little endosperm | Yes | PY |  |
| *Luzula spicata* (L.) DC. | Juncaceae | broad | starchy endosperm | No | PD | Deep |
| *Minuartia verna* (L.) Hiern subsp. *verna* | Caryophyllaceae | peripheral | conspicuously^d^ | No | PD | Deep |
| *Nardus stricta* L. | Poaceae | lateral | fleshy endosperm | No | PD | Deep |
| *Pedicularis tuberosa* L. | Orobanchaceae | linear underdeveloped | fleshy endosperm | No | MPD |  |
| *Phleum rhaeticum* (Humphries) Rauschert | Poaceae | lateral | starchy endosperm | No | ND |  |
| *Phyteuma hemisphaericum* L. | Campanulaceae | n.a | fleshy endosperm | No | PD | Non deep |
| *Phyteuma orbiculare* L. | Campanulaceae | n.a | fleshy endosperm | No | PD | Non deep |
| *Pilosella officinarum* Vaill. | Asteracea | spatulate fully developed | no endosperm genus | No | ND |  |
| *Poa alpina* L. | Poaceae | lateral | starchy endosperm | No | ND |  |
| *Polygala alpina* (DC.) Steud. | Polygalaceae | spatulate fully developed | fleshy endosperm | No | ND |  |
| *Potentilla aurea* L. | Rosaceae | spatulate fully developed | non-starchy endosperm | No | PD | Intermediate |
| *Primula farinosa* L. | Primulaceae | linear fully developed | hard or firm endosperm | No | PD | Non deep |
| *Pulsatilla alpina* L. | Ranunculaceae | rudimentary | fleshy endosperm | No | MPD | Intermediate complex |
| *Pulsatilla vernalis* (L.) Mill. | Ranunuculaceae | rudimentary | fleshy endosperm | No | MD |  |
| *Ranunculus montanum* Willd. | Ranunculaceae | linear underdeveloped | fleshy endosperm | No | PD | Deep |
| *Rhinanthus glacialis* Personnat | Scrophulariaceae | spatulate underdeveloped | fleshy endosperm | No | PD | Deep |
| *Scorzoneroides helvetica* (Mérat) Holub | Asteracea | spatulate fully developed | no endosperm | No | PD | Non deep |
| *Senecio doronicum* (L.) L. | Asteracea | spatulate fully developed | no endosperm | No | PD | Non deep |
| *Serratula tinctoria* L. | Asteracea | spatulate fully developed | no endosperm | No | PD | Non deep |
| *Sesleria caerulea* (L.) Ard. | Poaceae | lateral | starchy endosperm | No | ND |  |
| *Soldanella alpina* L. | Primulaceae | linear fully developed | hard or firm endosperm | No | PD | Non deep |
| *Solidago virgaurea* L. | Asteracea | spatulate fully developed | no endosperm | No | PD | Non deep |
| *Valeriana montana* L. | Valerianaceae | spatulate fully developed | no endosperm | No | PD | Deep |

ND= non dormant, PD=physiological dormancy; MD= morphological dormancy; MPD=morphophysiological dormancy; PY=physical dormancy

^a^ All from Baskin and Baskin (2007)

^b^ All from Martin (1946)

^c^ All from Baskin et al., 2000

**Figure S1.** Final germination percentage (FGP) of both habitats under the two incubation temperatures and treatments **a.** Incubation temperature 15/5° C and 0° C. **b.** Incubation temperature 25/15°C. Significant (p>0.05*)* differences indicate in different letters.

**
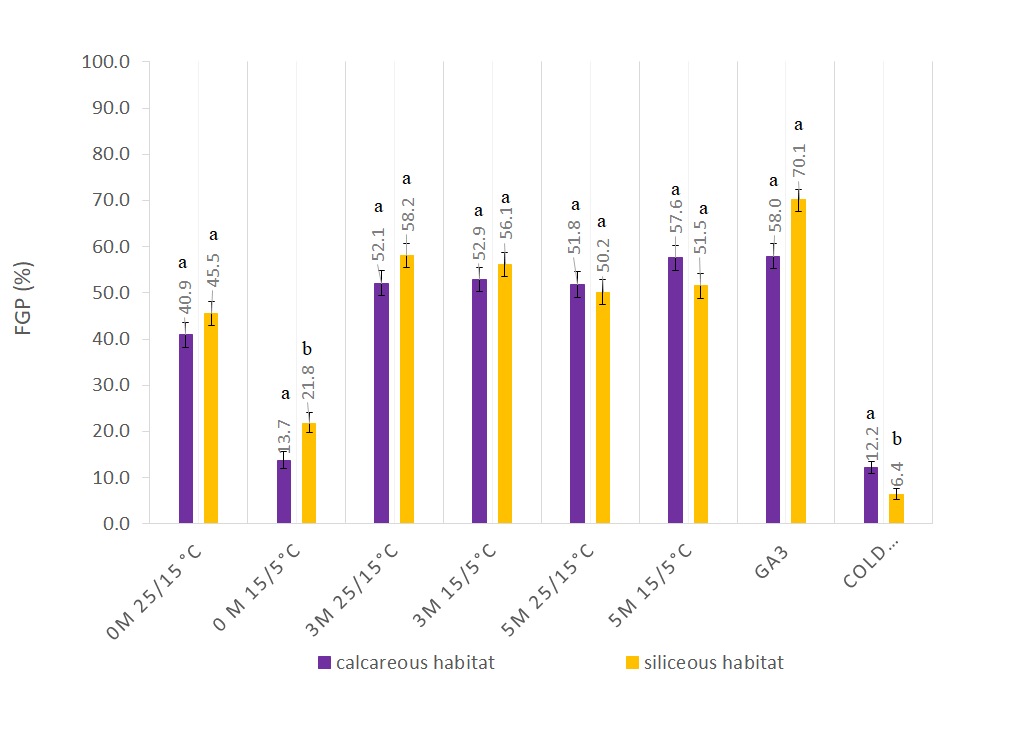
**

**Table S4*.*** Results and variable factor map of the PCA to defined germination traits. The first table represent the eigenvalues and the second the correlation between the variables and the Axis I and II. Finally, there is a graphic representation of the variable factor map from the PCA.

| **Eigenvalues** | **Correlation** | |
| --- | --- | --- |
|  | Axis I | Axis II |
| Variance | 7.49 | 2.07 |
| % of variance | 57.65 | 15.99 |
| Cumultative variance | 57.65 | 73.64 |

*Correlations.*

| **Variables** | **Correlation** | |
| --- | --- | --- |
|  | Axis I | Axis II |
| Cold stratification | 0.170 | 0.041 |
| 0CS-15/5 | 0.455 | -0.777 |
| 3CS-15/5 | 0.929 | 0.210 |
| 5CS-15/5 | 0.881 | 0.332 |
| 0CS-25/15 | 0.728 | -0.499 |
| 3CS-25/15 | 0.915 | 0.190 |
| 5CS-25/15 | 0.900 | 0.244 |
| T_50_ 0CS-25/15 | -0.687 | 0.481 |
| T_50_ 0CS-15/5 | -0.379 | 0.813 |
| T_50_ 3CS-25/15 | -0.818 | -0.157 |
| T_50_ 3CS-15/5 | -0.871 | -0.162 |
| T_50_ 5CS-25/15 | -0.821 | -0.112 |
| T_50_ 5CS-15/5 | -0.846 | -0.123 |


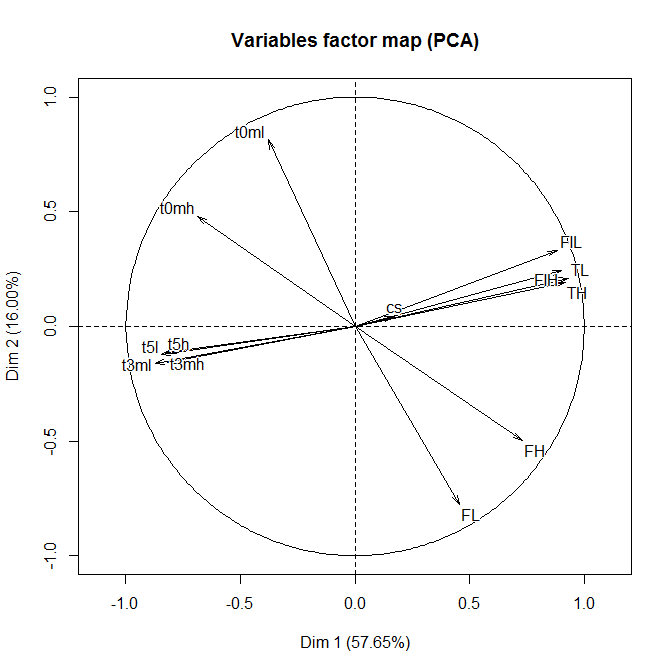


**Table S5a**. Results from the ANOVA tests assessing seed weight differences between habitats. Significant (P <0.05) values in bold character. **b.** Results from the lineal models correlating weight and Axis I, Aix II, FGP0M15/5°C and FGP0M25/15°C.

**a.**

| **Response variable** | | **λ** | | **AIC** | | **F-value** | | **p.value** | |
| --- | --- | --- | --- | --- | --- | --- | --- | --- | --- |
| Habitat | 0.36 | | 97.02 | | 0.017 | | 0.896 | |  |
| Microhabitat | 0.39 | | 98.83 | | 0.096 | | 0.908 | |  |

**b.**

| **Response variable** | | **Intercept** | | **Slope** | | **R squared** | | **p.value** | |
| --- | --- | --- | --- | --- | --- | --- | --- | --- | --- |
| Axis I | -0.443 | | -0.283 | | -0.016 | | 0.67 | |  |
| Axis II | 0.115 | | 0.078 | | -0.018 | | 0.83 | |  |
| FGP 0M 15/5°C | 0.148 | | -0.017 | | -0.018 | | 0.79 | |  |
| FGP 0M 25/15°C | 0.643 | | 0.061 | | -0.015 | | 0.63 | |  |
